# Deep Neural Networks for the Classification of Pure and Impure Strawberry Purees

**DOI:** 10.3390/s20041223

**Published:** 2020-02-23

**Authors:** Zhong Zheng, Xin Zhang, Jinxing Yu, Rui Guo, Lili Zhangzhong

**Affiliations:** 1 National Research Center of Intelligent Equipment for Agriculture, Beijing 100097, China; zhonzheng9-c@my.cityu.edu.hk (Z.Z.); zhangx@nercita.org.cn (X.Z.); yujx@nercita.org.cn (J.Y.); guor@nercita.org.cn (R.G.); 2 School of Data Science, City University of Hong Kong, Hong Kong, China; 3 Key Laboratory for Quality Testing of Hardware and Software Products on Agricultural Information, Ministry of Agriculture, Beijing 100097, China

**Keywords:** adulteration detection, deep neural networks, fruit purees, GRU, LSTM, TCN

## Abstract

In this paper, a comparative study of the effectiveness of deep neural networks (DNNs) in the classification of pure and impure purees is conducted. Three different types of deep neural networks (DNNs)—the Gated Recurrent Unit (GRU), the Long Short Term Memory (LSTM), and the temporal convolutional network (TCN)—are employed for the detection of adulteration of strawberry purees. The Strawberry dataset, a time series spectroscopy dataset from the UCR time series classification repository, is utilized to evaluate the performance of different DNNs. Experimental results demonstrate that the TCN is able to obtain a higher classification accuracy than the GRU and LSTM. Moreover, the TCN achieves a new state-of-the-art classification accuracy on the Strawberry dataset. These results indicates the great potential of using the TCN for the detection of adulteration of fruit purees in the future.

## 1. Introduction

The adulteration of fruit purees or juices has long been a serious problem that needs to be carefully considered by manufacturers. This problem arises frequently out of two main reasons. On one hand, the adulteration of fruit purees or juices is profitable since certain fruits command premium prices. For instance, a variety of fruits, such as apple, raspberry, blackcurrant, blackberry, plum, cherry, apricot and grape, are usually used to adulterate strawberry purees. These fruit purees are always cheaper than strawberry purees and could be mixed with strawberry purees to make impure strawberry purees. Impure strawberry purees are then sold as pure strawberry purees for higher profits. On the other hand, the direct detection of the adulteration is quite difficult because of the nuance of flavor and color of pure and impure strawberry purees [1]. To deal with the problem of adulteration detection, a number of quality control methods have been employed, such as high-performance liquid chromatography (HPLC), thin layer chromatography (TLC) enzymatic tests (e.g., sorbitol), and physical tests (e.g., pH) [2]. However, these extensive chemical analyses are always time-consuming and expensive [1], which motivates the adoption of spectroscopic techniques for detecting the adulteration. [3,4,5] have successfully utilized the Fourier transform infrared (FT-IR) spectroscopy to screen a number of adulterants in a range of food products. Specifically, the mid-infrared spectrum is commonly used for the adulteration detection of fruit purees [6].

The adulteration detection of fruit purees based on spectroscopy is a time series classification (TSC) problem of two classes: the authentic fruit and the adulterated fruit. Based on spectroscopy, various conventional algorithms have been employed for the adulteration detection of fruit purees. These methods include partial least square regression (PLS) [1], dynamic time warping (DTW) [7], random forest (RF) [8], rotation forest (RotF) [9], etc. Reference [10] compared dozens of conventional algorithms for the adulteration detection of strawberry purees and found that the RotF achieved the best classification accuracy. However, one impediment of conventional methods is they always require manually designed feature extractors, which are usually labor consuming and require specific domain knowledge.

During the last few decades, deep neural networks (DNNs) have recently achieved great success in a number of time series modeling tasks [11,12,13,14] and motivate the recent utilization of deep learning models for TSC [15]. Contrary to conventional methods, the biggest advantage of DNNs is the feature extraction could be conducted by the neural network automatically. Two major neural network architectures, Recurrent Neural Networks (RNNs) and Convolutional Neural Networks (CNNs), are commonly adopted for time series analysis. For the RNNs, a number of advanced variants, such as the Long Short Term Memory (LSTM) [16] and the Gated Recurrent Unit (GRU) [17], have been proposed to mitigate the vanishing gradient problem of RNNs due to training on long time series. Among different convolutional architectures, the temporal convolutional network (TCN) has been shown to achieve a comparable performance than RNNs in a number of sequential modeling tasks [18,19,20,21]. TCN is a convolutional network specially designed for sequence modeling tasks. The authors of references [19,20] proposed the basic TCN and the advanced TCN, TrellisNet, which achieved state-of-the-art classification accuracy on the Sequential MNIST, Permuted MNIST and Sequential CIFAR-10 datasets. The authors of references [18,21] developed two different variants of the TCN, the stochastic TCN and Wavenet, for the sequence generation.

Motivated by the great success of DNNs, this paper first employs DNNs for the adulteration detection of fruit purees and provides a performance comparison of different DNNs. Three types of DNNs—GRU, LSTM, and TCN—are tested on the Strawberry dataset from the UCR time series classification repository [22]. Experimental results demonstrate that TCN performs best among different DNNs in terms of the classification accuracy on the test dataset. Also, TCN achieves the new state-of-the-art classification accuracy on the Strawberry dataset. This result demonstrates the great potential of using TCN for the adulteration detection of fruit purees.

## 2. Materials and Methods

In this section, the publicly accessible dataset, Strawberry dataset, from the UCR time series classification repository is first introduced. The training criterions, evaluation metrics, as well as the training descriptions of three deep neural networks for TSC are then described in detail. Next, the network structures of the GRU, the LSTM and the TCN for TSC are introduced in detail, respectively.

### 2.1. The Strawberry Dataset

The Strawberry dataset contains the mid-infrared spectra of 983 fruit purees, including strawberry purees and non-strawberry purees, such as apple purees and plum purees. This dataset is a time series classification dataset of two classes—strawberry samples and non-strawberry samples. This dataset has been obtained using Fourier transform infrared (FTIR) spectroscopy with attenuated total reflectance (ATR) sampling. Each sample in the Strawberry dataset is a one-dimensional time series of length 235. For all the DNNs, each time series sample of length 235 is fed into the model as a two-dimensional input of size 1 × 235. The statistics of the Strawberry dataset are shown in Table 1.

### 2.2. Training Criterions and Evaluation Metrics

Given the true label $p\in {\left\{0,1\right\}}^{C}$ and classification Bernoulli vector $q\in {\left[0,1\right]}^{C}$, the cross entropy loss used to train the DNNs for TSC is shown in Equation (1):

(1) $$L_{H}=-\sum_{c=1}^{C}\left[p_{c}logq_{c}+\left(1-p_{c}\right)log\left(1-q_{c}\right)\right],$$

where C=2 denotes the total number of classes in the classification problem.

The classification accuracy on the test dataset is used to evaluate the model performance. The formal definition of classification accuracy is shown in Equation (2),

(2) $$classification\;accracy=\frac{N_{r}}{N}\times 100\%,$$

where $N_{r}$ and N denote the number of correctly classified samples and the total number of samples in the dataset.

### 2.3. Training Descriptions

The TCN in the experiment consists of six residual blocks. The dilation factors, *d*, and the filter size, *k*, of the dilated causal convolution are $d=2^{i}$ for layer i and k=6 for all layers in TCN. The dropout rate used in the TCN is set to 0. Both the GRU and the LSTM are made up of three recurrent layers. To keep approximately the similar size of GRU, LSTM, and TCN, the number of hidden units for the GRU and the LSTM are set to 64 and 56, respectively. In this way, the number of parameters of all DNN models is set to approximately 60,000 to ensure a comparable model complexity. The number of channels of each dilated causal convolution in the TCN is set to 32. All three models are trained by the Adam optimizer with the initial learning rate 0.0001, via minimizing the cross entropy loss between the true label and the predicted classification vector. A grid search is conducted to obtain the best output dropout rate. The total number of training epochs of all models is set to 1000.

In addition, the five-fold cross validation is conducted to find the optimal hyperparameters of all DNNs. Specifically, the output dropout rate varies in the set {0.0, 0.1, 0.2}. The optimal output dropout rate is selected based on the average classification accuracy on the five validation sub datasets. After the five-fold cross validation, the whole training dataset is used to train the DNNs with the best hyperparameters and the classification accuracies of all models are reported on the test dataset.

### 2.4. GRU and LSTM for TSC

Both GRU and LSTM are two popular variants of basic RNNs that incorporate gate units into the basic RNN cell to capture the long time dependency in the time series. In the GRU, two gate units, a reset gate $r_{t}$ and an update gate $z_{t}$, are introduced to control the transformation of hidden state $h_{t}$ at each time t. The control process of hidden states in the GRU is described in Equations (3)–(6):

(3) $$r_{t}=\sigma \left(W_{r}x_{t}+U_{r}h_{t-1}+b_{r}\right),$$

(4) $$z_{t}=\sigma \left(W_{z}x_{t}+U_{z}h_{t-1}+b_{z}\right),$$

(5) $${\tilde{h}}_{t}=\tan h\left(Wx_{t}+U\left(r_{t}⨀h_{t-1}\right)+b\right),$$

(6) $$h_{t}=\left(1-z_{t}\right)⨀h_{t-1}+z_{t}⨀\;{\tilde{h}}_{t}.$$

As another variant of the RNN, LSTM adopts three gate units, the forget gate $f_{t}$, the input gate $i_{t}$ and the output gate $o_{t}$, to control the cell state $c_{t}$ and hidden state $h_{t}$ at each time t. The updating of hidden states in LSTM is presented in Equations (7)–(11):

(7) $$f_{t}=\sigma \left(W_{f}x_{t}+U_{f}h_{t-1}+b_{f}\right),$$

(8) $$i_{t}=\sigma \left(W_{i}x_{t}+U_{i}h_{t-1}+b_{i}\right),$$

(9) $$o_{t}=\sigma \left(W_{o}x_{t}+U_{o}h_{t-1}+b_{o}\right),$$

(10) $$c_{t}=f_{t}⨀c_{t-1}+i_{t}⨀\tan h\left(W_{c}x_{t}+U_{c}h_{t-1}+b_{c}\right),$$

(11) $$h_{t}=o_{t}⨀\tan h\left(c_{t}\right).$$

For the TSC problem, the predicted classification vector $q\in {\mathbb{R}}^{C}$, where C denotes the total number of classes, is computed by adding a fully connected layer (FC) on top of the hidden state $h_{T}$ at the last time step T:

(12) $$q=W_{q}h_{T}+b_{q}.$$

**Figure 1:**
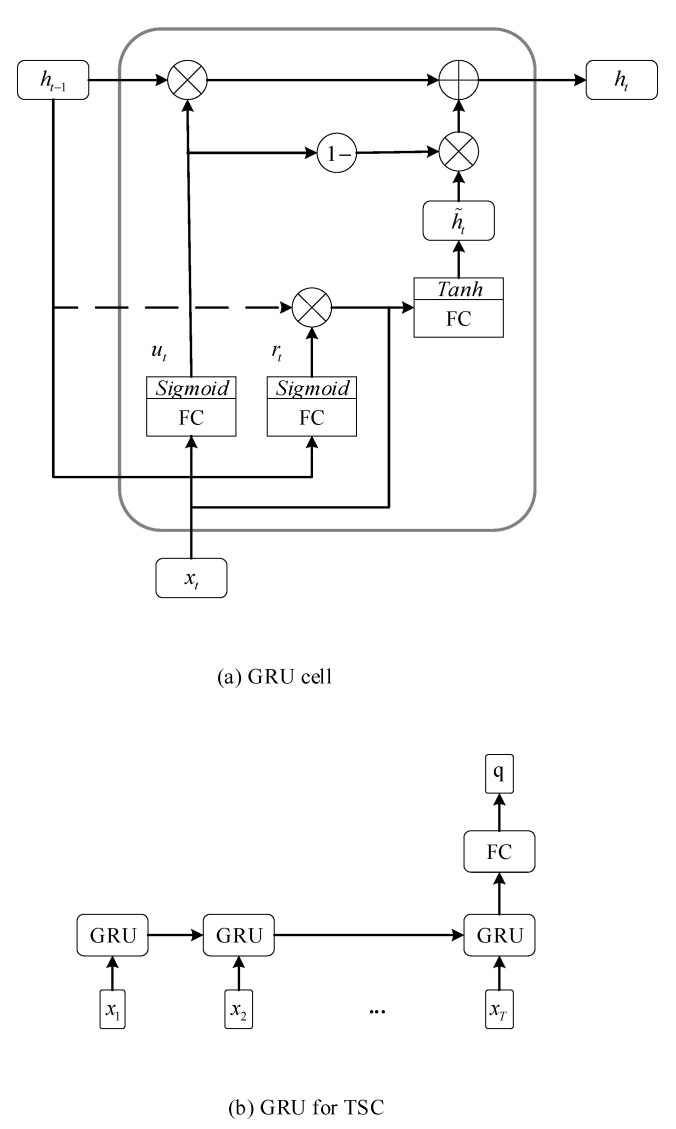
The schematic of the Gated Recurrent Unit (GRU) for time series classification (TSC).

**Figure 2:**
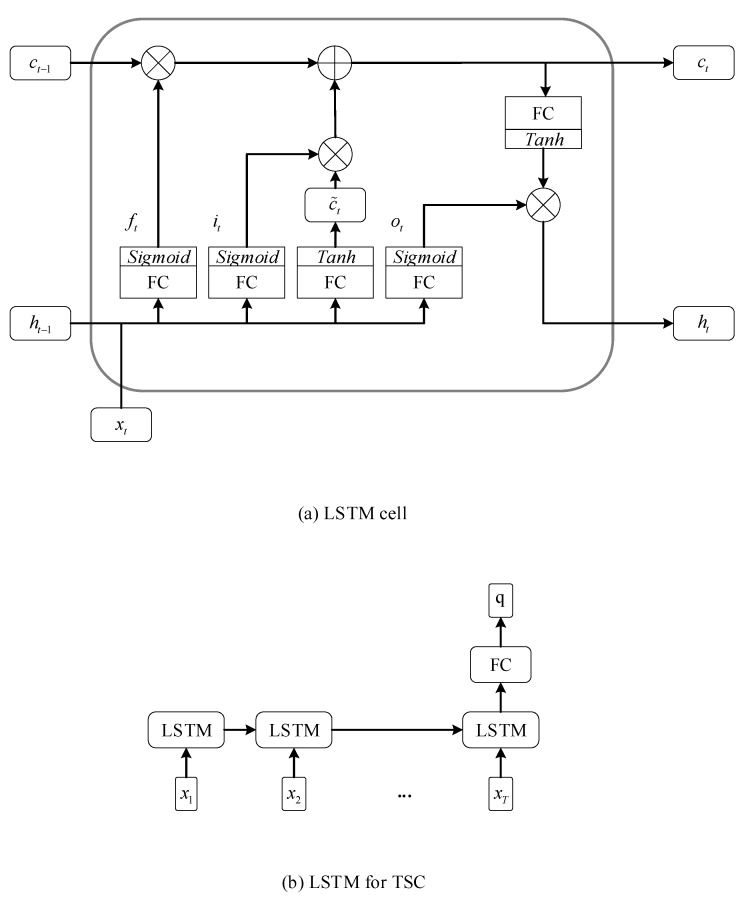
The schematic of the Long Short Term Memory (LSTM) for TSC.

In Equations (3)–(12), matrices, W, U, $W_{r}$, $U_{r}$, $W_{z}$, $U_{z}$, $W_{f}$, $U_{f}$, $W_{i}$, $U_{i}$, $W_{o}$, $U_{o}$, $W_{c}$, $U_{c}$, and $W_{q}$ as well as vectors, b, $b_{r}$, $b_{z}$, $b_{f}$, $b_{i}$, $b_{o}$, $b_{c}$, and $b_{q}$, are parameters which need to be optimized. The σ and ⨀ are utilized to denote and element-wise sigmoid function and the element-wise multiplication, respectively. The tanh represents the hyperbolic tangent function which serves as the activation function. The schematics of GRU and LSTM for TSC are shown in Figure 1 and Figure 2, respectively.

### 2.5. TCN for TSC

TCN is a specially designed convolutional network for sequence modeling. Formally, given an input sequence $x=\left(x_{1},x_{2},\ldots ,x_{T}\right)$, the output sequence $h=\left(h_{1},h_{2},\ldots ,h_{T}\right)$ is of the same length as the input sequence. Specifically, the TCN ensures that at each time step t, the output $h_{t}$ depends only on those inputs from the past: $x_{1},x_{2},\ldots ,x_{t}$. In other words, there is no information leakage from the future at any time. For the purpose of equivalent input and output length, the TCN adopts the 1D fully convolutional network (FCN) architecture [23]. In the FCN, each hidden layer has the same length as the input layer, and the zero padding of length (kernel size −1) is added to keep subsequent layers with the same length as previous ones. In order to prevent information leakage from the future, the TCN employs causal convolutions [19], rather than the normal convolutions. In the causal convolution, an output at time t is convolved only with elements from time t and earlier in the previous layer. In addition, the TCN adopts dilated convolutions [21] to enable an exponentially large receptive field. Different from normal convolutions, dilated convolutions introduce a fixed step between every two adjacent filter taps. Formally, given a 1-D sequence input $x\in {\mathbb{R}}^{n}$ and a filter f: {0,…k−1}→ℝ, the dilated causal convolution operation F on the element s of a sequence x is defined as

(13) $$F\left(x_{s}\right)=\left({x*}_{d}f\right)\left(s\right)=\sum_{i=0}^{k-1}f\left(i\right)\cdot x_{s-d\cdot i},\;\;x_{\leq 0}≔0$$

(14) $$h_{1},h_{2},\ldots ,h_{T}=F\left(x_{1}\right),F\left(x_{2}\right),\ldots ,F\left(x_{T}\right),$$

where d is the dilation factor, k is the filter size, s−d·i counts the number of directions from previous nodes to the current node, and h is the output sequence. Furthermore, TCN also employs the residual block [24], which has repeatedly been utilized to facilitate the construction of very deep networks.

**Figure 3:**
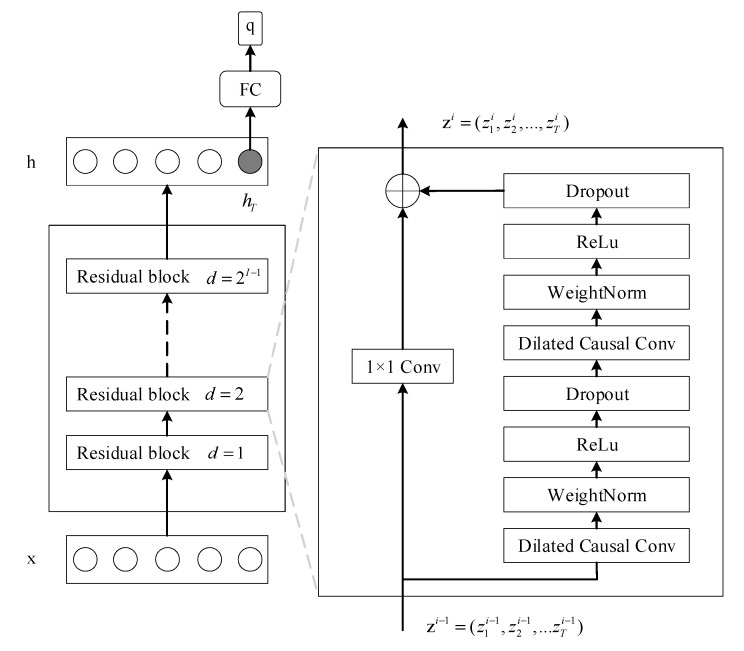
The schematic of the temporal convolutional network (TCN) for TSC.

As shown in Figure 3, TCN is constructed by stacking multiple residual blocks. Each residual block consists of two dilated causal convolutions. A fully connected layer is added on top of the last layer at the last time step to output the predicted classification vector.

## 3. Results and Discussion

**Figure 4:**
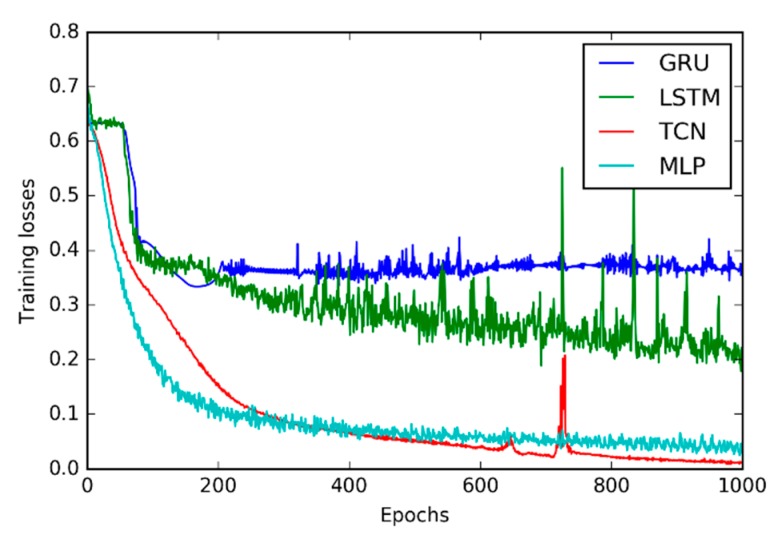
Training losses of the GRU, LSTM, TCN, and MLP.

The training losses of the GRU, LSTM, TCN, and Multilayer Perceptron (MLP) are illustrated in Figure 4. The comparison of classification accuracy of these three DNNs on the test dataset is then provided in Table 2. Table 3 shows the comparison of the training time of these four DNNs.

From Figure 4, it is observable that the TCN converges to lower classification loss than the GRU, LSTM and MLP.

Table 2 shows that the classification accuracy of the GRU and the LSTM is lower than the RotF by [10] as well as the TCN and MLP. This might result from the gradient vanishing problem of the recurrent architectures. On the other hand, the TCN obtains the highest classification accuracy among all the models. The TCN obtains the state-of-the-art classification accuracy of 98.65% on the Strawberry dataset. This result indicates the great potential of using the TCN for the detection of the adulteration of fruit purees.

Table 3 shows the comparison of the training time of different DNNs. It is observable that the training of MLP is much faster than the GRU, LSTM and TCN. Furthermore, the training of TCN is much faster than the GRU and LSTM.

## 4. Conclusions

In this paper, a comparative study of DNNs for the classification of pure and impure strawberry purees was provided. Specifically, three different types of DNNs—GRU, LSTM, and TCN—were implemented for the detection of the adulteration of strawberry purees. These three models were tested on the Strawberry dataset from the UCR time series classification repository. Computational experiments indicated that the TCN obtained a higher classification accuracy than GRU, LSTM, and MLP. Also, in comparison to the best accuracy, 97.30%, obtained by the conventional algorithm RotF [10], the TCN achieved a new state-of-the-art classification accuracy of 98.65% on the Strawberry dataset. These results indicate that it is promising to use the TCN for the detection of the adulteration of fruit purees in the future.

## Figures and Tables

**Table 1:** Statistics of the Strawberry dataset.

Dataset	Number of Samples
Training dataset	613
Test dataset	370

**Table 2:** Classification accuracy (%) of different models on the test dataset.

Model	Classification Accuracy
RotF [2]	97.30
MLP	96.76
GRU	90.54
LSTM	87.84
TCN	**98.65**

**Table 3:** Training time (in seconds) of different models.

Model	Training Time
MLP	**41.44**
GRU	253.03
LSTM	250.81
TCN	131.58
